# The shift from old age to very old age: an analysis of the perception of aging among older people

**DOI:** 10.1186/s12875-021-01616-4

**Published:** 2022-01-11

**Authors:** Emile Escourrou, Sarah Laurent, Jacques Leroux, Stéphane Oustric, Virginie Gardette

**Affiliations:** 1 Département Universitaire de Médecine Générale, Faculté de Médecine Rangueil – Université Paul Sabatier Toulouse III, 133 route de Narbonne, 31400 Toulouse, France; 2 Maintain Aging Research team, CERPOP, Université de Toulouse, Inserm, Université Paul Sabatier, Toulouse, France; 3 Maison de Santé Pluriprofessionnelle Universitaire La Providence, Toulouse, France; 4 Centre Hospitalier Universitaire de Toulouse, Toulouse, France

**Keywords:** Age 80 and over, Aging, Life change events, Independent living, Longevity, Qualitative research

## Abstract

**Background:**

The oldest-old (individuals over 90 years) are a fast-growing population. Understanding the perceptions of older people about very old age is the first step towards developing optimal geriatric care for an aging population. This study aimed to explore the potential shift from old age to very old age through the exploration of older people’s perception of aging.

**Methods:**

Qualitative study conducted through individual interviews in the homes of older people. We voluntarily chose to include persons a decade under and above 90 years old to explore other factors than age that could participate in the shift from old age to very old age. The sampling was theoretical. We carried out the analyses using an inductive approach based on the phases of grounded theory. The researchers used triangulation. Collection was concluded when theoretical saturation was reached.

**Results:**

Fourteen participants were interviewed. The shift from old age to very old age was not based on age but occurred when participants became conscious of the irreversibility of aging and its effects, and when they started living day-by-day, renouncing to any plan in a near future. The transition to very old age seemed to be preceded by a progressive disengagement from non-essentials activities. Participants reported a sensation of progressive social exclusion due to the loss of contemporaries or spouse, the difficulty to connect with younger generations or the absence of relationships in their neighborhood. The last step of life was feared, not because of the idea of death itself but because of the associated suffering and loss of autonomy.

**Conclusion:**

Precipitating and slowing factors of the shift to very old age were identified to help general practitioners support older patients throughout their life trajectories.

## Background

In the care of older people, a new population of patients is growing: the oldest-old. The American Geriatric Society and the World Health Organization define the oldest-old as individuals aged over 80 years, while the British Geriatrics Society uses 85 years as a threshold. In recent publications, the cut off was fixed at 85 or 90 years and over [1–3].

According to worldwide forecasts, the population of oldest-old is likely to triple in the next 30 years, from 126.5 million to 446.6 million [4]. This trend can be explained by changes in socio-environmental factors and health behavior. Factors such as diets, the presence of a caregiver, economic status, are correlated with better aging [5–10]. Regarding biological factors, genetic signatures could be correlated with long-living individuals [6, 9].

In this population of oldest-old, the aim of individual care is to allow successful aging at home by preventing disability and loss of abilities [11, 12]. The desire to age at home, as well as population projections, create challenges for health care organizations, particularly in primary care.

General practitioners (GPs) are involved in the care of older patients, from the prevention of disability to end-of-life care [13, 14]. One of the goals of GPs is to provide a patient-centered approach based on deep respect for patients as unique individuals, and the obligation to care for them on their terms [15]. GPs adapt guidelines at their disposal to the unique situation of the patient and his/her environment.

In order to adapt guidelines, a better knowledge of the characteristics of this population is needed: can we consider the oldest-old in the same way than “classic” older patients? Recent studies aimed to describe the cognitive, physical, functional and nutritional status of the oldest-old, providing data on the global characteristics of this population [16–20]. To better understand the characteristics of the oldest-old, it is also necessary to explore their own perception. Understanding the perceptions of the oldest-old is the first step towards developing optimal geriatric care for an aging population [21]. Future research on changes of self-perceptions of aging will provide insights into the mechanisms of resilience of the aging self in later life [22].

The experience of aging is influenced by personal and environmental factors. This experience depends on the person’s health status and their perception of their health status [23], and in a more holistic way, on their intrinsic capacity [24]. The environment, in the broadest sense, influences the experience of aging. The access to neighbors, convenience stores, public transport, health care facilities, family members, are all factors that influence the experience of aging [25]. The position of older people in the society, depending on the local culture and the place of living, also has a major impact on the perception of aging among older people [26, 27].

These individual (intrinsic) and environmental (extrinsic) factors can be explored through a life course approach. Life course approach is defined by the entirety of an individual’s life and the circumstances an individual experience while aging [28]. The approach recognizes that all stages of a person’s life are intricately intertwined, not only with each other, but also with the lives of family members (past, present and future) and other people in society [29]. Thus, it takes a temporal and societal perspective of the health of individuals and acknowledges that health and well-being depend on interactions between risk and protective factors throughout people’s lives [29]. Complementary to this approach, the aging trajectory (part of the life course) could be summarized as what people foresee for themselves and the process by which they construct and revise their subjective aging trajectory [30].

As factors of old age and aging are now well known, we could hypothesis that they are also major factors in the perceptions of very old age. To our knowledge, the life course approach and the aging trajectory haven’t yet been the subject of a qualitative study to explore older people’s perceptions of very old age. Our research question was: is there a continuum or a significant shift from old age to very old age in the perceptions of older people?

This study aimed to explore a potential shift from old age to very old age through the exploration of older people’s perception of aging.

## Methods

We undertook a qualitative study through semi-structured interviews of older people carried out between September 2018 and May 2019.

### Population and recruitment

Three recruitment sources were used: general practitioners, medical and social action coordination centers, hospitals in north- and south-west France. Recruitment was carried out by snowball sampling to reach older people not necessarily included in medical or social care settings. We used diverse recruitment sources to obtain as varied a sample as possible. Participants were recruited according to the technique of theoretical sampling [31, 32].

Individuals included in the study were living at home in north-west and south-west France. We voluntarily chose to include persons a decade under and above 90 years old for two main purposes: (1) to better explore a potential discontinuity in perceptions from old age to very old age through the description of the participants’ life course, (2) to explore the hypothesis that age is not the only factor that influences the feeling of belonging to the oldest-old population. For this purpose, we chose to include persons also a decade under and above 90 with various profiles regarding the characteristics of interest: marital status, presence of children, place of living, living environment, involvement of domestic help/nurses, relationship with neighbors, chronic disease(s). The choice of these characteristics of interest originated in literature on personal and environmental factors influencing the experience aging, summarized in the background section. We hypothesized that those characteristics influence a potential shift from old age to very old age.

We did not retain in the sample persons who had cognitive disorders or who were unable to give their consent.

After each interview, a new participant was selected to obtain a different profile. After the recruiter obtained the participant’s consent, the researcher carrying out the interviews (SL or JL) contacted the participant by telephone. The researcher introduced himself as carrying out a study on aging. The interviews were all carried out in the homes of the older persons by the researchers (SL or JL), general practitioners trained in semi-directive one-to-one interview methods. Recruitment was carried out over 7 months.

### Sample

Fifteen interviews were conducted. Fourteen allowed to reach theoretical saturation. One interview was excluded from the analysis as the participant was more severely cognitively impaired than was supposed by the recruiter. The interviews lasted an average of 50 min. The participants were aged from 79 years to 97 years old. The mean age was 89.3 years. The sample contained 7 women and 7 men. The characteristics of the study population are summarized in Table 1.

**Table 1:** Characteristics of participants interviewed

Individuals	Age (years)	Marital status	Children	Place of living, Living environment	Domestic help / Nurses	Relationship with neighbors	Chronic disease(s)	Recruitment sources ^**a**^
Mrs. D	90	Widow	2 sons	Home, urban	+/−	–	+	GP
Mrs. F	92	Widow	1 daughter	Home, urban	+/−	–	–	GP
Mrs. T	84	Married to Mr. T	2 sons	Home, urban	−/−	+	–	GP
Mr. T	90	Married to Mrs. T	2 sons	Home urban	−/−	+	+	GP
Mr. B	92	Widower	1 son, 1 deceased daughter	Home, urban	+/−	+	–	GP
Mr. R	89	Married	1 daughter, 1 deceased son	Home, rural	+/+	–	+	H
Mr. D	93	Widower	None	Residential home for older people, urban	+/+	–	+	GP
Mrs. C	87	Widow	1 son	Home, urban	+/−	+	–	SC
Mr. L	79	Married	1 son	Home, rural	+/+	–	+	GP
Mr. Ro	88	Married	3 daughters, 1 son	Home, urban	+/+	–	+	GP
Mr. LB	95	Single	None	Nursing home, urban	+/−	+	–	Snowball sampling
Mrs. L	94	Widow	2 sons	Home in the ownership of her daughter, rural	+/+	+	–	Snowball sampling
Mrs. Th	80	Widow	2 daughters	Home in the ownership of her daughter, rural	+/−	+	+	Snowball sampling
Mrs. R	97	Widow	1 daughter, 1 deceased daughter	Home, semi-rural	+/+	–	+	Snowball sampling

^a^*GP* General Practitioner, *H* Hospital, *SC* Social center

### Data collection

Semi-directive one-to-one interviews were conducted, and the research physicians (SL or JL) also recorded direct open observations. Observations of the participant’s home and environment were carried out before, during, and after the interview. The observation was non-participative, indirect, undisguised and unstructured. The results of observations allowed to contextualize the data collected from speech. All participants signed a written consent form before audio recording began. The transcriptions from audio recording to text support were anonymized. An evolving two-part interview guide was used: 1) situation of the participant (age, life situation, living environment, illnesses), exploration of his/her life course; 2) perception of their aging trajectories and expectations regarding the support required for better aging. Data collection was concluded when theoretical saturation was achieved.

### Data analysis

Empirical data (transcriptions, audio recordings, logbooks of observational data) were analyzed using an inductive approach based on the different phases of grounded theory [31, 32].

#### First step

The first step was verbatim transcriptions into open code, based on the transcription of speeches and the contextualization from observations (EE, SL, JL). Each verbatim idea was translated into an open code. A single sentence could lead to different open codes if several ideas were revealed.

#### Second step

The second step was the creation of conceptual categories (EE, SL, JL). This stage started after the third interview. Conceptual categories were created by combining open codes resulting from the same concept. A title was given to each conceptual category created after an effort of conceptualization starting from the global idea.

#### Third step

After creating the conceptual categories, we elaborated patterns and linkages between them to create themes that seemed relevant to answer the research question. Each theme was based on the combinations of parts of conceptual categories. From those themes, one appeared as an emergent principal theme and the others as secondary themes (EE, SL, JL).

#### Fourth step

The theorization was the elaboration of a theoretical explanatory model which answered the research question. It was based on the explanation of the linkages between the principal theme and the secondary themes, reinforced by to-ing and fro-ing between empirical and theoretical data. We did not use specific analysis software. The resulting theory was influenced by the theory of symbolic interactionism: the study of the microsociological environment and the interactions between individuals through the effect of their mutual behavior [33].

Throughout these stages, triangulation was carried out by two other research physicians (EE, SL, JL). Triangulation was used to illuminate blind spots in an interpretive analysis.

### Ethics

The study was carried out in accordance with relevant guidelines and regulations, namely the French legislation of March 5, 2012 (law 2012–300 known as Jardé law) amended on June 16, 2012 (amendment 2016–800).

The study was validated by the ethics committee of the French National College of Teachers of General Practitioners under the number 14031965B (17 April 2019).

## Results

Four themes were identified after analysis. The major theme was the description of “the stage of very old age”. This was the stage described by the participants as the next stage of aging after old age. This stage was not based on age as in the literature but was defined when a participant acknowledged a progressive and uncontrollable decline.

The other themes identified were the progressive disengagement from activities during the transition to very old age; the progressive exclusion from the surrounding environment and the resignation of the older people; and finally, the fear of the last stage of life. To construct their perceptions of aging, and the transition from old age to very old age, participants confronted their actual perception of their situation, with the stage they expected to be in considering their life course and the surrounding world. We described those four themes and linked them to one another to suggest a theory to answer our research question.

### Disengagement in their activities during the transition to very old age

Participants progressively adapted to their experience of aging. They gradually reduced their level of activity: shorter driving distances, lower frequency and duration of activities, more local travel destinations. In some cases, a total elimination of activities was described. These changes took place more or less abruptly depending on the circumstances.

The intrinsic factors influencing this disengagement were: physical condition (reduced vision or hearing, reduced mobility and physical strength, illness) or psychological condition (loss of vital impulse). Extrinsic factors were also found: children restricting their parents’ activities due to concern for their safety, death of contemporaries, modification of the couple’s balance when their spouse died or their health deteriorated (illness necessitating regular care). The loss of a spouse was identified as a major factor that influenced negatively several parts of their fragile daily balance, from loss of vital impulse and sadness, to difficulty in maintaining daily activities previously done by the spouse (shopping, cooking, management of personal finances, etc.). When changes in the participant’s situation were unpredictable, the changes seemed imposed. For example, participants did not feel the same way if they stopped driving after a stroke (unpredictable change) versus if they decided to stop driving because they felt gradually less confident. *“I used to love reading and painting, but now... I still read a bit with a magnifying glass, but painting, museums and all that, I don’t see well enough, so, hum, my life has narrowed a bit” Mrs. D.* Even if this disengagement was regretted, it seemed to be chosen to reduce risks and conserve the energy needed to maintain important activities giving meaning to life. Participants progressively agreed to delegate more tiring tasks and chose to prioritize decisional autonomy over organizational autonomy.

At the opposite, we identified certain slowing factors of this disengagement, especially extrinsic factors. Aging participants were grateful for help from family members, as long as relationships remained balanced. The couple was considered as a solid and faithful support system, but its disappearance could bring about a destabilizing change. A network of professional caregivers could also allow to maintain contact and support. The continuation of social relationships and shared activities gave meaning to life. Regarding intrinsic factors, a preserved health status gave a solid basis to maintain activities. *“I have everything I need! I could not wish for better. I have my children nearby, my grand-children are adorable and come to visit me, it is enough”. Mrs. L.*

### The stage of very old age: acknowledging a progressive and uncontrollable decline

*“It came progressively, but I find that it happens quickly now. Mrs. L.*

Before the stage of very old age, old age appeared as a slow, progressive and often unconscious process. It wasn’t felt to match official age, although some key ages were recognized *“The 90 years threshold” Mrs. D.* It seems difficult to be aware of the process. The participants did not know whether to define themselves as old. This insidious evolution was regretted as it could not be prevented. *“No, getting old I can’t say, I don’t think I became aware of it! That’s what’s bad about it!” Mr. B.* Age progression was perceived as bringing incapacities and disabilities.

Then, the stage of very old age was identified when the effects of biological aging affected lifestyle, and it became difficult or impossible to maintain activities. At this stage aging was keenly felt. The shift to very old age, identified after analysis, was not based on age. The shift occurred when older people became aware of the irreversibility of aging. This awareness led to an absence of project-making for the near future. It was felt as increased susceptibility to fatigue, lowering of physical or cognitive abilities, slowing motor skills, heightened anxiety. *“Oh it was, it took place progressively, I don’t know, one year, two years that I can’t do what I did before. Before I was doing the garden, I was helping, now I can’t anymore” Mrs. D.*

When the capacities of aging subjects diminished and they needed to be assisted for common activities (shopping, preparation of meals, hygiene, mobility etc.), dependency appeared and could be a revealing factor of very old age. Loss of autonomy for transport and in particular the abandonment of driving seemed a decisive step, as persons were deprived of a freedom of action. *“After all we have everything, all around us. We have the, we go to buy apricots in such a place, you used to go to small villages, so if you don’t have the car you can’t go anymore. So that’s why he doesn’t want to go there anymore” Mrs. T.* In this case, an important wish was to keep a hold on the management of their house with the bare necessities to live in their own place as long as possible.

### A progressive exclusion from the surrounding environment and resignation

*“I think from a certain point in time you should know how to be invisible or at least very discrete” Mr. LB*

Advancing age was accompanied by a limitation of social relationships due to the loss of one’s contemporaries who passed away or drifted away. *“In the village too, the elderly go either to the cemetery or to the retirement home” Mrs. D.* The social network of contemporaries in the neighborhood could be replaced little by little by a younger generation, not in tune with the older one, reinforcing the feeling of isolation. With restricted mobility, the home often became the center around which socializing was organized. To keep social links, some attempted to build relationships with their younger neighbors, others forced themselves to move further to meet people. Home carers could be expected to play the role of a social connection. Radio or television allowed to keep a contact with the changing exterior world. *“So, well I’m happy, I speak with them for two hours, they don’t do much, they don’t have much know-how either” Mrs. C.*

An inversion of family dynamics was reported. The participants progressively lost their decisional autonomy while their children decided for them, sometimes imposing a restriction of their activities. Recognizing the family network as essential during the aging process, they accepted a submissive position, tried to become invisible and avoided being a burden for their children to preserve this relationship. *“My children conspired to make me understand that it was time that I gave up my freedom” Mrs. L.* In time, relationships could loosen, excluding the older people who felt they were becoming less important. Distance from family members increased the feeling of isolation. *“The isn’t much else besides New Year and Christmas... and a few small family occasions, which are always interesting but one feels a bigger and bigger distance” Mr. LB.* In the absence of descendants, loneliness was even more marked in very old age. The consciousness of the end of a family line could be painful. *« Alone! Alone alone it’s despairing, despairing… Being alone, being alone… Not even the telephone, the telephone doesn’t ring, nothing. » Mrs. C.*

A feeling of indifference and negligence of society towards older people was experienced. The feeling of being useless, of becoming a weight, or even a danger, was expressed, and could bring the subjects to want to withdraw. Older people considered that today’s society was adapted to the younger generation and not to them. This place apart from the rest of society could be difficult to accept. *“Young people don’t care” Mr. L.*

### The feared last stage

*“THE question, question without answer: WHERE, WHEN, HOW? [...] I’m scared about how it will happen, that is, for example to be frank, of suffering: will it be calm, serene? Or will it be in illness, in suffering, in short how it will unfold” Mrs. D.*

Age progression and diminishing individual capacities placed the subject in a situation of fear and incertitude towards the future. The future seemed narrowed down and the time remaining to live unknown. The participants expressed a certain fatality towards the future, projecting a constant and uncontrollable worsening of their situation (worsening of dependency, suffering illness, loss of activities and so on). *“I think the future is the home stretch and it’s the one with the most hurdles and it’s the one that can end up with bad news” Mr. R.* The evolution towards dependency was associated with the fear of becoming a weight or a burden for close ones, and the risk of institutionalization. Participants adopted a guarded position towards the future, perceiving that at this age, the situation can shift rapidly. *“So, what can you do? Tomorrow I’m going to do my shopping and I wonder every time if it isn’t the last time, because...” Mrs. C.*

The question of death took a larger place. Death was described like an inevitable and uncontrollable phase towards which people were getting nearer as they aged. Death was often perceived as the next stage in life and appeared imminent. Progressive loss of their contemporaries reinforced the inexorability of death and reminded participants that their turn was getting nearer. *“One runs, one flies towards what will take place finally” Mr. R.* By realizing their finitude, individuals could come to feel that they were running out of time, leading them to give up certain obligations. Projects were culled until they disappeared completely. The future was perceived as a close continuation of the present, until it disappeared completely. *«Well, I have the project to continue I don’t know how, but I don’t make big projects at the moment. I live day by day” Mr. LB*. Some could find hope in religion, putting their future in the hands of God. *“Make the most of the present moment. Little things, family life, family meals, a sunny day when one is walking with family or friends, et cetera. All these things of daily life live them intensely. Because it’s the present that counts for us, not the future, the present” Mrs. D.*

### Towards a theory of the shift from old age to very old age

**Fig. 1:**
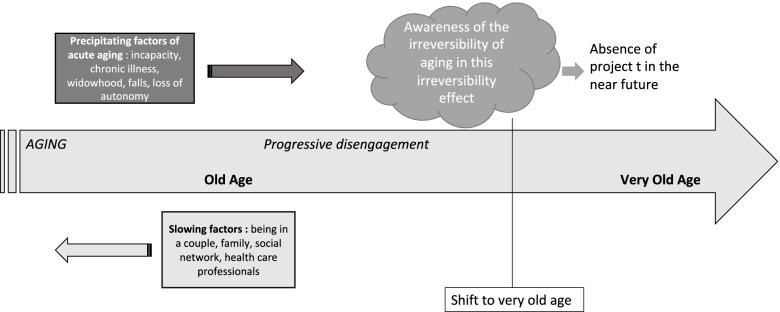
Theorization of the step to very old age

The results described above led us to attempt to theorize the shift to very old age (Fig. 1). This attempt to theorize illustrates the links between the themes described above.

In our study, the progression towards the stage defined as very old age was described as insidious. It could be precipitated by certain factors such as becoming incapacitated, health issues, falls, dependency, isolation, widowhood. At the opposite, being in a relationship, having a family and/or social network, the involvement of health care professionals, were identified as slowing factors of the insidious progression to very old age.

The shift towards very old age, as defined after analysis of participants’ interviews, took place when one became conscious of the irreversibility of aging. Capacities to adapt seemed overwhelmed. Aging became synonymous with uncontrollable decline. In this downward spiral, the future narrowed, became uncertain. The relationship with time changed, and projects lost their place. At this stage, participants preferred to live day by day, made the most of the present moment.

## Discussion

### Summary of results

Participants identified a progressive and insidious process of aging during their life course. The diminution of their physical and mental abilities forced a disengagement in their activities to keep their energy for the activities answering an absolute need for their daily living. A progressive exclusion from the surrounding world and social connections was also identified: loss of contemporaries (spouse in particular) and inversion of family and social dynamics with the younger generation. Our analysis supported a shift from old age to very old age not based on age but occurring when subjects became conscious of the irreversibility of aging and its effects, and when they started living day-by-day, renouncing to any plan in a near future. They feared the last stage, projecting a constant and uncontrollable worsening of their situation.

### Intrinsic and extrinsic factors of aging during life course

The disengagement theory was proposed in the early twentieth century in US social science [34]. Similar intellectual currents were observed in other countries. In early 2000, S. Clément and J. Mantovani suggested a definition of disengagement: the process of reorganizing life according to physical and relational changes emerging during one’s life course [35]. We found this concept useful to describe the participants’ choices during their aging trajectories and the way they decided where to concentrate their energy.

We hypothesized that age was not the only criteria to define very old age. Participants mentioned chronological age as one variable that brings the consciousness of aging, but not the major one. To explore more in depth the impact of age during a person’s life course, it is necessary to differentiate chronological age and perceived age, and better understand why the gap narrows between these two concepts when nearing very old age [36]. The use of perceived age seems to have a closer correlation with older people’s capacities: for example, perceived age is better correlated with cognition status than chronological age [37].

The inversion of family dynamics was a sign of this step into very old age. When disability occurs, compromises need to be made to adapt one’s daily organization, and children’s choices take a larger place [38]. Participants identified this change as potentially difficult. Inversion of family dynamics also influenced the children and family caregiver’s lives, as it can be associated with different type of feelings from solidarity to conflict [39]. The role of family caregiver has long been identified as negatively affecting one’s everyday life [40, 41] as caring for one’s aging family member could be seen as a burden [42]. In a holistic approach, the GP as a “family doctor” may assess the familial dynamics and propose specific intervention for older people and their caregivers. Interventions for caregivers go from psychological support, to education on the parent’s disease, to targeted prevention against caregivers neglecting personal care [43].

The reduction of social relationships, the loss of contemporaries, widowhood, the generational gap associated with loss of mobility, were factors confining older people to their home. Even if successful aging involves staying in one’s home [44], isolation becomes more and more important and can turn to loneliness [45]. A friendly neighborhood and the presence of informal helpers in the local environment permit to avoid this feeling of loneliness [46, 47].

From participants speeches, it appeared that they weren’t generally afraid of death, as they were aware and resigned to their age status. In our GP practice, it could be difficult to differentiate this resignation from an authentic depression when facing an older patient for whom death is an important subject of discussion [48]. Asking older patient near the shift to very old age about their wishes for end of life and death could help improve patient-centered care.

### Implications for care

To help improve patient centered care, the approach advocated by the World Health Organization of intrinsic capacity seems adapted to the need identified in our study [49]. The preservation of fundamental capacities: cognitive, psychological, locomotive, energetic, sensory, has to be the principal and global aim of caregivers, not just the care of each pathology separately [21].

This approach seems adapted to prevent the shift to very old age as defined in our study, but also to mitigate its effects if the shift has already happened. A pluri-professional approach appears necessary to identify and manage the precipitating and slowing factors of a shift to oldest age (Table 2). Precipitating factors and slowing factors were identified in the different themes described in the Results section. We propose a summary in this table to better identify these factors.

**Table 2:** Precipitating and slowing factors of a shift to oldest age

Dimensions	Precipitating factors	Slowing factors
Physical health	Acute disease Physical incapacity Loss of autonomy	Satisfactory health condition Preservation of mobility
Mental health	Loss of vital momentum Willing of death Lack of projects in the near future	Maintenance of activities of daily living
Social environment	Widowhood Insufficient social network Social isolation	Presence of family members, friends, Neighbors in the environment Cohabitation Appropriate social measures

The care plan has to be centered on patients’ wishes and priorities especially regarding their social environment and intrinsic capacities [50]. Non-pharmacological approaches such as social and physical activities have their place in a care plan to maintain intrinsic capacities [51].

Although it is clear that the oldest-old are in the last part of their life course, prevention should still be a significant aspect of global care and needs further research.

### Strengths and limitations

This study explores a growing topic justified by the demographic evolution of western societies. The qualitative method with the choice of an analysis based on the grounded theory allowed a comprehensive approach of the perception of the participants. This factor can then be considered in GP care plans for their older patients. The rigor of the methodology was assured first by the theoretical sampling method, then through the analyses conducted, with an inductive approach fulfilling the step of the grounded theorization, and by researchers’ triangulation [52].

The relatively small number of interviews was balanced by the in-depth approach and the richness of the discussions.

The researchers didn’t have precise information regarding the health status of participants besides those given by the recruiters and the participants themselves. A cross-analysis of the functional status assessed by health professionals and the information collected during the interview could have been useful to understand further the situation of the participant.

The age of the researchers (around 30 years old) could have influenced the relationship between the researchers and the participants. Participants could have a familiar approach towards the researcher projecting a grandfather role during the interview. To prevent the establishment of such relationship dynamics, the researcher who realized the interview was empathic but neutral and did not talk about his personal point of view or his personal life course.

## Conclusion

The oldest-old are a fast-growing population worldwide. The aim of individual care is to allow successful aging at home by preventing disability and loss of abilities. The desire to age at home, as well as population projections, create challenges for health care organizations, particularly in primary care. Better understanding older people’s perceptions of very old age could help to adapt their care plan to their own experience. This study aimed to explore a potential shift from old age to very old age through the exploration of older people’s perception of aging.

Participants identified a progressive and insidious process of aging during their life course. The diminution of their physical and mental abilities forced a disengagement from non-vital activities. A progressive exclusion from the surrounding world and social connections was also identified. The shift from old age to very old age was not based on age but occurred when subjects became conscious of the irreversibility of aging and its effects, and when they started living day-by-day, renouncing to any plan in a near future.

The shift to very old age could be precipitated by certain factors such as becoming incapacitated, health issues, falls, dependency, isolation, widowhood. At the opposite, being in a relationship, having a family and/or social network, the involvement of health care professionals, were identified as slowing factors of the insidious progression of aging into old age. Precipitating and slowing factors of the shift to very old age were identified and could help general practitioners support older patients with a care plan throughout their life trajectory.

## Data Availability

The data analyzed during the current study are available from the corresponding author on reasonable request.
